# Magnetic Field-Guided Magnetic Nanoparticles as Neurotherapeutics for Neurological Disorders and Glioblastoma

**DOI:** 10.3390/life16020293

**Published:** 2026-02-09

**Authors:** Ming-Chang Chiang, Yu-Ping Yang, Christopher J. B. Nicol

**Affiliations:** 1 Department of Life Science, College of Science and Engineering, Fu Jen Catholic University, New Taipei City 242, Taiwan; 2 Sylvester Comprehensive Cancer Center, Miller School of Medicine, University of Miami, Miami, FL 33136, USA; yyang22@med.miami.edu; 3 Department of Biochemistry and Molecular Biology, Miller School of Medicine, University of Miami, Miami, FL 33136, USA; 4 Departments of Pathology & Molecular Medicine and Biomedical & Molecular Sciences, and Cancer Biology and Genetics Division, Sinclair Cancer Research Institute, Queen’s University, Kingston, ON K7L 3N6, Canada; nicolc@queensu.ca

**Keywords:** magnetic field, magnetic nanoparticles, exosome–MNP complexes, Alzheimer’s disease, Parkinson’s disease, stroke, glioblastoma

## Abstract

Neurodegenerative diseases, including Alzheimer’s disease (AD), Parkinson’s disease (PD), and stroke, are among the most devastating neurological disorders worldwide. Glioblastoma (GBM) is a rapidly growing cancer that originates in astrocytes in the brain. It invades and damages the nervous system. Current treatment options remain limited, primarily due to poor blood–brain barrier penetration, lack of targeted delivery, and limited efficacy in slowing disease progression or promoting functional recovery. In recent years, magnetic fields (MFs) have emerged as a promising therapeutic approach, with mechanisms of action that include direct neuromodulation and the guidance of magnetically responsive nanocarriers to the lesion. Magnetic nanoparticles (MNPs), owing to their unique magnetic properties, biocompatibility, and responsiveness to external MFs, have emerged as promising therapeutic agents for the treatment of neurological diseases and glioblastoma. Exosome–magnetic complexes combine biological carriers with magnetic responsiveness to enhance targeting and biocompatibility for the treatment of neurological diseases and glioblastoma. This review highlights recent advances in magnetic field- and MNP-based neuroprotective strategies and explores new methods for targeted intervention and translational research using exosome–MNP complexes.

## 1. Introduction

Neurodegenerative diseases—including Alzheimer’s disease (AD), Parkinson’s disease (PD), and stroke—remain among the most devastating disorders of the nervous system, with limited disease-modifying options [1,2,3]. AD is characterized by extracellular amyloid-β (Aβ) plaques and intracellular hyperphosphorylated tau tangles, which lead to synaptic loss and disruption of the hippocampal–cortical network [4,5]. PD is characterized by pathological Lewy bodies that contain high concentrations of α-synuclein and by loss of dopaminergic neurons in the substantia nigra, leading to motor impairment [6,7]. Ischemic stroke occurs when there is a sudden reduction in cerebral blood flow due to thrombosis or embolism, which can lead to excitotoxicity, thrombotic inflammation, and nerve damage [8,9].

Glioblastoma (GBM), although not a neurodegenerative disorder, is the most common and aggressive primary malignant brain tumor in adults and causes profound neurological disability and mortality [10,11,12]. GBM exhibits significant heterogeneity, immunosuppression, and challenges related to blood–brain barrier (BBB) delivery, which contribute to resistance and recurrence [13,14].

We include GBM in this review because magnetic fields (MFs) and magnetic nanoparticle (MNP)-enabled strategies share key translational constraints across degenerative and neoplastic CNS diseases (e.g., BBB penetration, target localization, and the need for non-invasive spatiotemporal control) [15,16].

MFs have emerged as a novel therapeutic modality that may address these unmet needs. Depending on their frequency and intensity, MFs can exert biological effects ranging from modulating oxidative stress and inflammation to promoting synaptic plasticity, neurogenesis, and cognitive changes [17,18,19]. Furthermore, when combined with magnetic nanoparticles or other magnetically responsive carriers, external MFs enable guided drug delivery and multimodal imaging [20,21,22]. This dual function as both a direct therapeutic tool and an enabling technology positions MFs as a promising frontier in neuromedicine.

MNPs are engineered nanoscale materials (typically ~5–200 nm) composed of a magnetic core and a stabilizing/functional surface coating, rather than single molecules [23,24]. In biomedical applications, the core is most commonly an iron oxide (e.g., magnetite, Fe_3_O_4_, or maghemite, γ-Fe_2_O_3_). At the same time, the shell (e.g., dextran, PEG, silica, or lipid/polymer coatings) improves colloidal stability, reduces aggregation and nonspecific protein adsorption, and provides chemical handles for conjugating targeting ligands and therapeutic cargo [25,26]. Many clinically oriented formulations are designed to be superparamagnetic, exhibiting strong magnetization under an applied field but minimal remanence after field removal, which reduces irreversible particle clustering.

MNPs have emerged as promising candidates in this context, offering several unique advantages over traditional approaches [27,28]. Owing to their intrinsic magnetic properties, MNPs can be precisely guided to diseased regions of the brain through the application of external MFs, thereby improving the localization of therapeutic agents [29,30]. In addition, MNPs can be engineered to carry a wide range of cargo, including drugs, small interfering RNAs (siRNAs), genes, and antioxidant molecules, enabling versatile therapeutic applications [31,32,33]. Beyond their drug-delivery potential, MNPs also serve as diagnostic tools, functioning as magnetic resonance imaging (MRI) contrast agents [23,34,35]. This dual capability positions MNPs as theranostic platforms, combining diagnostics and therapeutics to provide integrated, personalized approaches to treating neurodegenerative diseases.

## 2. Properties of MFs and MNPs

Several modalities of magnetic field exposure have been studied in preclinical and clinical settings. Static magnetic fields (SMFs) and other complex time-varying magnetic fields (e.g., rotating, pulsed, or low-frequency paradigms) have been reported to influence redox balance, mitochondrial activity, and inflammatory pathways [36,37,38,39]. Pulsed electromagnetic fields (PEMFs) penetrate tissue and modulate time-varying fields in cell signaling; they are widely used in bone repair and are increasingly being used for neurodegeneration [40,41]. Low-frequency magnetic stimulation (LFMS) enhances neuronal excitability and synaptic plasticity, with emerging data in depression and cognitive impairment [42,43]. When combined with MNPs, externally applied MFs can enhance spatiotemporal control over particle distribution and local retention, potentially improving delivery to target brain regions under BBB-constrained conditions, while also enabling image-guided assessment of biodistribution and target engagement [44,45,46]. Collectively, these MF–MNP modalities provide a flexible toolkit that can be tailored to disease context, therapeutic payload, and required depth/precision of targeting across CNS indications.

MNPs exhibit a range of physicochemical properties that make them attractive for biomedical applications, particularly in the treatment of neurodegenerative diseases [47,48,49,50,51]. The most widely studied materials include magnetite (Fe_3_O_4_) and maghemite (γ-Fe_2_O_3_), which are valued for their strong magnetic response and relatively low toxicity [52,53,54]. Other compositions, such as cobalt ferrite (CoFe_2_O_4_) and manganese ferrite (MnFe_2_O_4_), provide enhanced magnetic performance but often raise additional safety concerns, underscoring the importance of balancing magnetic strength with biocompatibility To optimize their therapeutic utility, MNPs are commonly modified through surface functionalization. Coatings such as polyethylene glycol (PEG) improve solubility and circulation time, while conjugation with antibodies, peptides, or aptamers allows for targeted delivery to specific neuronal or glial cell populations [55,56,57]. More recently, hybrid platforms combining MNPs with biological carriers, including exosomes, have been developed to enhance biocompatibility further, reduce immune clearance, and facilitate efficient passage across the BBB [58,59]. In practical terms, MNPs are nanoscale magnetic cores (most commonly iron oxides, e.g., magnetite/maghemite), often stabilized by polymer, silica, or lipid coatings that enhance colloidal stability, biocompatibility, and functionalization. Therapeutic use typically involves (i) engineering the MNP surface to carry or bind a payload (drug, gene, antioxidant, or protein), (ii) administering the formulation systemically or locally, and (iii) applying an external MF (static gradient for guidance/retention, or alternating MF for actuation such as hyperthermia-triggered release) while monitoring distribution with MRI or other imaging.

In terms of physical characteristics, MNPs often exhibit superparamagnetism at nanoscale dimensions, which allows them to respond to external MFs without retaining residual magnetization once the field is removed [24,60]. This property minimizes aggregation and reduces potential off-target effects. Particle size, typically 10–100 nm, plays a crucial role in determining biodistribution, stability in physiological environments, and the ability to penetrate the BBB [23,61]. Proper stabilization strategies are essential to prevent particle agglomeration and to ensure consistent in vivo performance. Biocompatibility and toxicity remain central considerations in the development of MNPs for clinical applications [62,63]. Although iron oxide nanoparticles (IONPs) are generally recognized as safe and have been approved for use as MRI contrast agents, issues such as oxidative stress, inflammation, and long-term accumulation must be carefully evaluated [62,64]. Surface coatings and functionalization strategies significantly influence biocompatibility by modulating interactions with plasma proteins, immune cells, and neuronal tissues [65]. Therefore, systematic assessment of toxicity, biodistribution, and clearance is critical for the successful translation of MNP-based therapies into clinical practice.

## 3. Mechanisms of Action in the Nervous System

Most mechanistic evidence discussed in this section is derived from preclinical studies (in vitro experiments and in vivo animal models), and many MF/MNP-based interventions remain investigational rather than routine clinical practice. Accordingly, reported effects should be interpreted in the context of model systems, exposure parameters, dose/field safety limits, and translational constraints (e.g., delivery depth, biodistribution, and target specificity). The biological mechanisms by which MFs may exert neurobiological effects have been investigated primarily in preclinical settings. For example, specific magnetic field exposure paradigms have been reported in in vitro and in vivo models to transiently modulate BBB permeability, thereby enhancing the delivery of co-administered agents to the central nervous system [66,67]. Magnetic stimulation has also been associated (in model systems) with modulation of ion channel activity, reductions in reactive oxygen species (ROS), and improvements in mitochondrial function [68,69]. In addition, external MFs can, in principle, guide nanoparticles, cells, or drug-loaded vehicles toward lesions for spatially biased targeting. However, the achievable depth and precision depend strongly on field geometry and tissue distance [23,70].

The therapeutic potential of MNPs in neurodegenerative diseases is supported mainly by in vitro experiments and animal studies in which magnetically responsive carriers are used to improve CNS delivery under external MF control [47,71]. One of the most critical barriers to effective treatment is the BBB, which restricts the passage of most pharmacological agents into the brain. In preclinical models, targeted MFs have been used to increase local retention or bias the distribution of MNP-associated payloads toward regions of neuronal injury or degeneration, thereby improving site-specific exposure while reducing systemic delivery requirements [47,59,72]. Once localized, MNPs can function as versatile carriers for a wide variety of therapeutic molecules. Drug delivery applications include encapsulating or conjugating antioxidants, anti-inflammatory agents, and compounds designed to inhibit amyloid-β (Aβ) aggregation, thereby addressing key pathogenic processes in AD [73]. Similarly, gene and siRNA delivery via MNPs has demonstrated potential to suppress the expression of pathological proteins such as tau, α-synuclein, and amyloid precursor protein (APP), thereby providing a strategy for targeted molecular intervention.

Beyond their use as pharmacological cargo, MNPs have been explored as tools to enhance cell-based therapies in preclinical studies. Magnetic labeling of stem cells (typically with IONPs) has been used in vitro and in vivo to enable external MF guidance and increase local cell retention in targeted CNS regions [57,74]. However, outcomes depend on the delivery route, field strength or gradient, and tissue depth, which enable external magnetic-field guidance [57,74]. This approach not only increases the engraftment efficiency but may also facilitate neuronal differentiation and tissue repair, offering opportunities for regenerative medicine in neurodegenerative disorders. Intracellular MNPs convert external MFs into mechanical cues that potentiate exosome biogenesis and secretion, simultaneously promoting nanoparticle hand-off between cells [75]. These findings suggest that magneto-mechanical control is a general strategy for amplifying exosome yields, tuning cargo composition, and programming nanoparticle distribution in nanomedicine. A further advantage of MNPs is their capacity to serve as multifunctional platforms for multimodal diagnosis and treatment. IONPs can act as contrast agents for MRI, enabling non-invasive monitoring of biodistribution and therapeutic efficacy [64,76]. When combined with drug or gene delivery, this dual functionality establishes MNPs as theranostic agents, bridging diagnostics and therapeutics within a single system [35,47]. Such integrated approaches hold promise for advancing precision medicine strategies in the management of neurodegenerative diseases.

## 4. Applications in Neurological Disorders and GBM

MF-based interventions span a continuum from clinically implemented neuromodulation to investigational magnetically enabled nanomedicine, and evidence of their maturity varies substantially across indications. In clinical practice, MF application is best established as transcranial magnetic stimulation (TMS) and related paradigms [77], delivered by positioning an external coil over defined cortical targets and administering repeated stimulation sessions with protocol-specified parameters (e.g., frequency, intensity, train structure) to modulate neural excitability. By contrast, most MF applications for neurodegenerative diseases and GBM remain at the research or trial stage, and reported benefits should be interpreted in the context of the model system [22,78,79]. Recent syntheses and mechanistic papers consolidate evidence that static and alternating MF (AMF) and frequency-dependent paradigms can achieve neuromodulation and targeted actuation in CNS models. MNPs have been increasingly investigated for their therapeutic and diagnostic potential across multiple neurodegenerative and neurological disorders [47,71,80]. By leveraging MFs’ guidance, targeted delivery, and multifunctional capabilities, MNP-based systems have shown promise in addressing disease-specific mechanisms that are otherwise difficult to modulate with conventional therapies [24,29].

**Figure 1:**
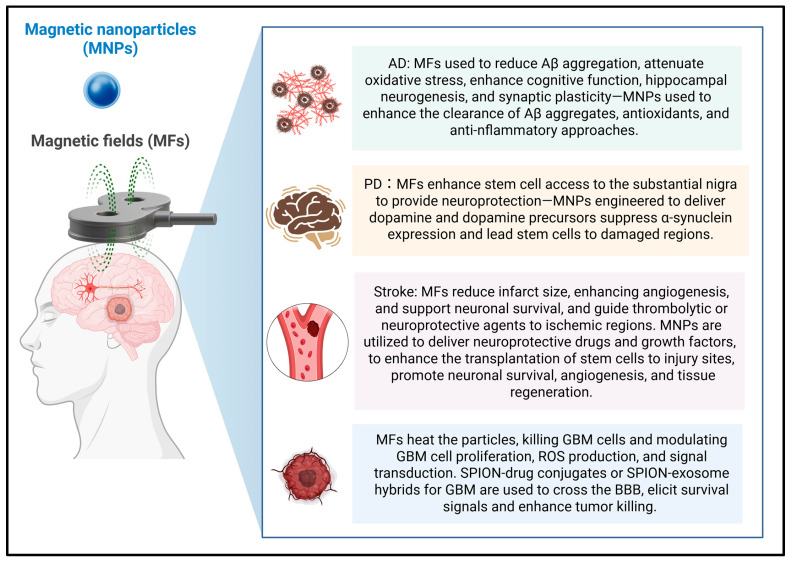
**MF-guided MNPs as neurotherapeutics in neurodegenerative disorders, stroke, and glioblastoma.** MFs can modulate biological responses and guide the accumulation of MNPs to targeted brain regions. In AD, MFs/MNPs may reduce Aβ aggregation and neuroinflammation and mitigate oxidative stress. In PD, magnetic guidance can enhance the delivery of dopaminergic therapeutics and support stem-cell homing and neuroprotection. In ischemic stroke, MF-guided MNPs enable localized delivery of thrombolytic, neuroprotective, or pro-angiogenic agents and can improve cell-based therapy targeting. In GBM, MF-mediated heating (magnetic hyperthermia) and MF-guided SPION-based drug and exosome hybrids may enhance BBB crossing, modulate ROS/signaling, and potentiate tumor killing. The figure was created in BioRender. Chiang, M. (2026). https://BioRender.com/glzi24d, accessed on 18 January 2026.

Based on the provided diagram (Figure 1) and current research in nanomedicine, this overview outlines how MNPs and MFs are being developed as a “dual-force” therapy for complex brain disorders. MFs: External magnets generate fields that can guide particles to a specific site in the brain or induce vibration or heating to trigger a biological response. MNPs: Tiny particles, often IONPs or Superparamagnetic IONPs (SPIONs), that can be coated with drugs, antibodies, or stem cells.

### 4.1. AD

AD is a progressive neurodegenerative disorder and the leading cause of dementia worldwide, characterized clinically by insidious onset and gradual deterioration of memory, executive function, and behavior [81]. Pathologically, AD features extracellular Aβ plaques and intracellular neurofibrillary tangles composed of hyperphosphorylated tau, accompanied by widespread synaptic loss, selective neuronal vulnerability, and network disconnection across hippocampal–cortical circuits [5]. In AD, the accumulation of Aβ plaques, oxidative stress, and neuroinflammation are central drivers of pathology. Despite decades of research, disease-modifying treatments remain limited. Recent anti-amyloid antibodies can reduce plaque burden and modestly slow clinical decline in selected patients, but therapeutic benefit varies and safety, access, and timing challenges persist [82]. Tau-targeted strategies, anti-inflammatory approaches, metabolic and mitochondrial modulators, and neuroprotective interventions are under active investigation [83].

Given the multifactorial nature of AD, there is an urgent need to integrate mechanism-based therapies with innovative strategies [2,84]. A recent meta-analysis of randomized clinical trials assessed the use of repetitive TMS (rTMS) for cognitive impairment in AD. This analysis summarized the features of the protocols used, including stimulation targets and parameters, as well as the observed cognitive outcomes. The findings indicated that rTMS was associated with improvements in cognitive measures compared with sham or control treatments [85]. Lopez-Barbosa et al. developed PEGylated magnetite nanoparticles for intracellular delivery of siRNA targeting BACE1, an important enzyme involved in Aβ generation [86]. The design incorporates elements to enhance cellular uptake and facilitate endosomal escape, such as co-immobilization strategies. This study exemplifies a methodology that combines a carrier with nucleic acid loading and assesses cellular delivery and biocompatibility endpoints. This approach supports claims that MNPs are a viable preclinical delivery method.

In this context, the development of magnetically guided MNPs as neurotherapeutic agents represents a scalable intervention for AD (Table 1). Magnetic field interventions have been shown to reduce amyloid-β (Aβ) aggregation, attenuate oxidative stress, and enhance cognitive function in animal models [87] and human brain tissue [88]. PEMFs may promote hippocampal neurogenesis and synaptic plasticity [89,90]. MNPs have been used to enhance the clearance of Aβ aggregates by magnetically guiding the delivery of anti-Aβ compounds, antibodies, and siRNAs targeting amyloid precursor protein (APP) [47,91]. In addition, MNPs functionalized with antioxidants and anti-inflammatory agents have demonstrated efficacy in reducing oxidative stress and attenuating neuroinflammation, thereby improving neuronal survival [47]. The combination of MNPs with imaging modalities also has potential for early diagnosis and real-time monitoring of therapeutic outcomes in AD models [21].

### 4.2. PD

PD is a progressive neurodegenerative disorder characterized by the formation of Lewy bodies and Lewy neurites within neurons [4,6]. These structures are primarily composed of misfolded α-synuclein [7]. Furthermore, selective degeneration of dopaminergic neurons in the substantia nigra pars compacta leads to striatal dopamine depletion [92]. Clinically, PD presents with bradykinesia, muscle rigidity, and resting tremor [93]. Dysfunction of neural circuits involves the basal ganglia-thalamus-cortical circuit, brainstem autonomic nuclei, and limbic system networks, resulting in heterogeneous phenotypes and disease progression trajectories [94]. Mechanistically, mounting evidence suggests that protein homeostasis disturbances (α-synuclein misfolding/aggregation and impaired autophagy-lysosomal flux), mitochondrial dysfunction and oxidative stress, calcium homeostasis imbalance, endoplasmic reticulum stress, neuroinflammation (microglia and stellate cell activation), and impaired axonal transport and synaptic maintenance are all associated with this disease [4,95,96]. Current treatments primarily aim to alleviate symptoms by restoring dopaminergic function (levodopa, dopamine agonists, MAO-B inhibitors, and COMT inhibitors) and by device-based interventions (deep brain stimulation of the subthalamic nucleus or the medial part of the globus pallidus; enteral levodopa infusion; focused ultrasound therapy for tremor) [97,98]. rTMS can enhance motor function in patients with PD, and improvements in gait and frozen gait have been observed in some studies [99]. However, the effectiveness of rTMS varies with the treatment regimen, including factors such as target selection (e.g., supplementary motor area (SMA) or primary motor cortex (M1)), treatment frequency, and per-session dose. Overall, rTMS demonstrates significant clinical application value. Niu et al. reported on multifunctional superparamagnetic nanoparticles that carry shRNA targeting α-synuclein [100]. This study was evaluated both in vitro and in vivo, with results focused on neuroprotection and repair in PD-relevant models. This approach is a strong example of how MNPs can be used effectively, as it links the platform, cargo, disease-related target, and in vivo outcomes while remaining in the preclinical stage.

Due to the complex and varied nature of PD, combining mechanism-based treatments with MF-guided MNPs as neurotherapeutic agents offers a promising strategy to enhance the management of the condition (Table 2). Low-frequency stimulation and MNPs-guided therapies have demonstrated protective effects on dopaminergic neurons and reduction in α-synuclein aggregation [47,101]. MFs may also enhance stem cell homing to the substantia nigra and neuroprotection in PD [102,103,104]. MNPs have been engineered for the controlled delivery of dopamine and dopamine precursors, enabling restoration of neurotransmitter balance with improved targeting and reduced systemic side effects [47,100]. Moreover, MNP-mediated siRNA delivery has been shown to suppress α-synuclein expression, potentially modifying disease progression. In regenerative applications, stem cells labeled with MNPs can be magnetically directed to damaged regions of the substantia nigra, enhancing neural repair and promoting functional recovery [74,105].

### 4.3. Ischemic Stroke

Ischemic stroke accounts for approximately 85% of all strokes and is caused by a sudden reduction in cerebral blood flow due to arterial thrombosis or embolism [106]. The most common causes are extensive vessel atherosclerosis, cardioembolism (e.g., atrial fibrillation), or small vessel fatty hyaline degeneration [8]. The subsequent ischemic cascade forms a metabolically inert infarct core surrounded by a salvageable ischemic penumbra [107]. Within minutes to hours, energy depletion triggers glutamate-mediated excitotoxicity, ion imbalance, mitochondrial dysfunction, oxidative stress, endoplasmic reticulum stress, and BBB disruption [108,109]. These events recruit peripheral immune cells and activate microglia and astrocytes, thereby exacerbating cytokine release, edema, and “thrombotic inflammation”, all of which collectively determine infarct size and neurological prognosis [9,110]. Focused magnetothermal brain stimulation using nanoparticles has been shown to enhance motor function recovery in a chronic-phase stroke rat model, providing a clear example of MF-enabled neuromodulation at the lesion site [111]. A study has shown that 10 daily sessions of 5 Hz rTMS targeting the ipsilateral M1, when combined with rehabilitation therapy, can enhance motor function recovery in patients with subacute stroke [112]. This study provides a clear treatment plan that includes daily stimulation, rehabilitation therapy, and specific motor endpoint indicators. This plan can be used to clinically regulate the recovery process, suggesting that the timing of treatment may be critical to success. Lin et al. linked recombinant tissue plasminogen activator (rtPA) to chitosan-coated MNPs and evaluated its targeting efficiency, thrombolytic effects, biodistribution, and potential toxicity for stroke treatment [113].

New research focuses on developing mechanism-based interventions and nanotechnology strategies, such as magnetic-field-guided magnetic nanoparticles, to target neuroprotective effects (Table 3). PEMFs and static fields have shown neuroprotective effects by reducing infarct size, enhancing angiogenesis, and supporting neuronal survival [114,115]. When combined with magnetic carriers, fields may guide thrombolytic or neuroprotective agents directly to ischemic regions, thereby accelerating tissue repair [116,117]. Beyond chronic neurodegenerative diseases, MNPs have also been investigated in acute neurological injuries such as ischemic stroke [118]. Using transgenic mouse models and a light-induced ischemic stroke paradigm (likely a photothrombotic stroke model), the study applies in vivo imaging and immunohistochemistry to track the biodistribution of MNPs and evaluate therapeutic effects [119]. Outcomes focus on thrombus dissolution and functional/neurological protection, supporting the delivery of MNPs as a promising approach for acute ischemic stroke intervention. In these contexts, MNPs have been utilized to deliver neuroprotective drugs and growth factors, as well as to enhance the homing of transplanted stem cells to injury sites [120]. These applications aim to promote neuronal survival, angiogenesis, and tissue regeneration [58]. The regenerative potential of MNP-assisted therapies highlights their versatility across both degenerative and injury-induced neurological conditions.

### 4.4. GBM

GBM is the most common and aggressive primary malignant brain tumor in adults [10,11]. Clinically, GBM presents with headache, seizures, focal neurological deficits, or progressive cognitive and behavioral changes, reflecting its rapid growth, diffuse infiltration, and tendency to invade vital brain regions [121,122]. MRI typically shows an irregular, contrast-enhancing mass with central necrosis and surrounding edema [123,124]. At the molecular level, GBM exhibits significant intertumor and intratumoral heterogeneity. This condition, coupled with a highly immunosuppressive, myeloid-rich tumor microenvironment and the protective effect of the BBB, is a fundamental cause of treatment resistance and treatment failure [13,14]. Current standard treatment regimens include maximal safe surgical resection followed by local radiotherapy, with adjuvant temozolomide (TMZ) [125,126,127]. Tumor-treating fields (TTFields) can prolong progression-free survival and overall survival in appropriately selected patients [128,129]. Despite these advances, median overall survival remains around 14–18 months, and almost all tumors recur [10,12]. Maier-Hauff et al. reported on the intratumoral administration of IONPs, followed by AMF-induced thermotherapy combined with radiotherapy in recurrent GBM, demonstrating feasibility and safety while reporting survival outcomes compared to conventional approaches [130]. Norouzi et al. developed biocompatible IONPs loaded with doxorubicin, positioning this method to overcome the BBB and multidrug resistance in GBM cells, while simultaneously achieving site-specific magnetic targeting therapy [131].

MFs and MNPs can provide a complex and diverse approach to addressing these challenges, and the following table outlines their therapeutic potential (Table 4). An external AMF (typically around 100–400 kHz) generates heat in the particles (approximately 41–45 °C), thereby killing GBM cells and making them more sensitive to radiotherapy/TMZ (RT/TMZ) [132,133]. The biological effects of static/low-frequency MFs may modulate the proliferation of GBM cells, reactive oxygen species (ROS), and signal transduction [134,135]. Using magnetic gradients to steer SPIONs-drug conjugates or SPION-exosome hybrids toward GBM, aiming to cross/avoid the BBB and concentrate payloads (TMZ, siRNA, miRNA, and CRISPR) in tumor or peritumoral niches [136,137]. Magnetic nanoparticles are closest to the clinic in GBM as intratumoral MHT (with MRI-guided delivery and CED to improve coverage) [138,139]. Experiences using intratumoral IONPs and AMF (NanoTherm) in GBM have reported feasibility and survival signals when combined with radiotherapy [130,140]. Lipid–magnetic TMZ constructs and multifunctional MNPs show enhanced tumor kill and controllable release under AMF in vivo models [141,142].

## 5. Future Directions, Perspectives, and Safety

The future of MNP-based strategies for neurodegenerative diseases will likely depend on integrating innovative delivery systems, advanced magnetic field technologies, and clinical translation pathways [47,143]. In the era of precision medicine, personalized magnetic field therapy represents another frontier. Tailoring magnetic field strength, gradient, and application protocols to individual patients could maximize therapeutic targeting while minimizing off-target effects. Such personalized strategies would also account for variations in brain anatomy, disease progression, and treatment response, advancing individualized interventions for complex disorders such as AD and PD. The development of novel non-invasive magnetic field control technologies is also crucial [22]. The integration of high-gradient MFs with TMS) offers opportunities to achieve deep-brain targeting without invasive procedures [144,145]. This approach could allow simultaneous neuromodulation and magnetically guided drug delivery, combining functional stimulation with molecular intervention. Finally, the clinical translation of MNP-based therapies requires a well-defined pathway from preclinical benchmarks to clinical trials. Standardization of nanoparticle synthesis, surface modification, and quality control will be essential to meet regulatory requirements.

Despite rapid progress, MNP-enabled interventions are associated with important limitations and potential complications that must be considered alongside efficacy [146,147]. Material composition and surface chemistry strongly influence biocompatibility: while iron-oxide-based formulations are often favored for translational work, alternative magnetic cores (e.g., cobalt-, nickel-, or manganese-containing ferrites) can raise additional concerns due to ion leaching and material-specific cytotoxicity, particularly under acidic or oxidative microenvironments. Even for IONPs, safety is not automatic; adverse outcomes may arise from dose-dependent oxidative stress, lysosomal overload, or disruption of iron homeostasis in susceptible tissues [148,149]. Systemically administered MNPs are frequently taken up by the mononuclear phagocyte system, leading to off-target accumulation in the liver and spleen and variable clearance kinetics [150,151]. This biodistribution can complicate repeat dosing and may increase the likelihood of immunological reactions, including cytokine responses [152]. In the CNS context, additional risks include microglial activation/neuroinflammation, unintended interactions with the neurovascular unit, and uncertainty about the extent to which signal changes reflect true parenchymal delivery rather than vascular/perivascular retention [47,153,154]. MF application introduces its own practical constraints. Static-gradient targeting is limited by achievable field gradients at depth, and “targeting” may primarily increase local retention rather than guarantee trans-BBB transport. For AMF-based applications, non-specific heating, thermal dose control, and patient/device constraints require careful management [155,156]. Heating depends on particle properties, local concentration, and tissue perfusion and may produce heterogeneous effects if the intratarget distribution is nonuniform (a key issue in GBM). Finally, translational applications depend on dose reporting, standardized characterization, and long-term safety assessments to support risk–benefit evaluations and clinical feasibility studies.

## 6. Conclusions

MFs hold strong potential as therapeutic agents for neurodegenerative diseases and stroke. Their unique ability to directly modulate cellular processes and indirectly enhance targeted drug delivery positions them as versatile tools in neuromedicine. While early studies support benefits in reducing oxidative stress and neuroinflammation and promoting regeneration, clinical translation requires addressing challenges in standardization, safety, and field control. With continued interdisciplinary research, magnetic field-based therapies may become integral components of precision medicine strategies for neurological disorders. MNPs represent a versatile and innovative therapeutic platform for addressing the complex challenges of neurodegenerative diseases. They can be guided by external MFs, enabling precise targeting of affected brain regions. Their multifunctional design allows for efficient drug and gene delivery and can be integrated with imaging technologies. This unique combination makes MNPs an up-and-coming therapeutic agent of localization, targeted therapy, and multimodal diagnostics. However, despite encouraging preclinical evidence, the successful translation of MNP-based treatments into clinical practice remains to be determined. Comprehensive studies are needed to address long-term safety concerns, optimize BBB penetration, and refine magnetic field control techniques. With continued interdisciplinary research, MNPs to advance the broader development of precision nanomedicine.

## Figures and Tables

**Table 1:** **MFs and MNPs as Neurotherapeutics in AD.** The table below summarizes the roles of MFs and MNPs as promising neurotherapeutics for AD. This approach targets the core pathologies of AD, including Aβ aggregation, oxidative stress, and cognitive decline.

Neurotherapeutic Approach	Field/Nanoparticle Strategy	Proposed/Observed AD-Relevant Effects	Evidence Model/System	Reference
Rotating magnetic field (RMF)	Non-contact RMF exposure	Inhibits Aβ aggregation; alleviates cognitive impairment	AD mouse model	[87]
Electromagnetic field stimulation (repeated exposure)	Repeated EMF stimulation	Lowers Aβ peptide levels	Primary human mixed brain tissue cultures	[88]
Targeted magnetic nanoparticles for Aβ detection	MNPs engineered for Aβ detection (magnetic targeting and signal readout)	Enables Aβ detection (supports early diagnosis and monitoring)	Targeted nanoparticle detection platform	[91]
Functionalized MNPs with anti-oxidant and anti-inflammatory agents	Surface-functionalized MNPs carrying antioxidants and anti-inflammatory payloads	Reduces oxidative stress and neuroinflammation; improves neuronal survival	Neurodegeneration-oriented nanoparticle therapeutic concept and applications	[47]

**Table 2:** **MFs and MNPs as Neurotherapeutics in PD.** The table below summarizes the roles of MFs and MNPs as neurotherapeutics for PD. The primary focus is on protecting the dopamine-producing dopaminergic neurons in the substantia nigra and reducing the aggregation of α-synuclein, a hallmark protein of PD.

Neurotherapeutic Approach	Field/Nanoparticle Strategy	Proposed/Observed PD-Relevant Effects	Evidence Model/System	Reference
Electromagnetic field (EMF) and SPION/IONP co-intervention	IONPs/SPIONs combined with EMF exposure	Neuroprotection of dopaminergic neurons: functional improvement signals in the toxin model	6-OHDA rat PD model	[103]
Transcranial pulsed electromagnetic field (tPEMF)	Noninvasive pulsed EMF stimulation	Improves quality of life measures	Human PD participants	[104]
EMF-assisted stem cell therapy	Mesenchymal stem cells (MSCs) are exposed and positioned within electromagnetic fields.	Enhanced therapeutic efficacy and neuroprotection	Rat PD model	[102]
MNP-enabled RNAi against α-synuclein	Multifunctional MNPs loaded with α-synuclein RNAi plasmid	Suppresses α-synuclein-related pathology; supports neuroprotection and behavioral rescue endpoints	PD model	[100]
Functionalized MNPs (multi-cargo, anti-oxidant and anti-inflammatory add-ons)	Surface-functionalized MNPs enabling combined neuroprotective and delivery functions	Supports dopaminergic neuron survival by mitigating oxidative stress and neuroinflammation	Multiple model types	[47]

**Table 3:** **MFs and MNPs as Neurotherapeutics in Ischemic Stroke.** The table below summarizes the roles of MFs and MNPs as neurotherapeutics for acute neurological injury, specifically Ischemic Stroke. The core strategies involve neuroprotective, reduction in infarct size, and promotion of tissue regeneration.

Neurotherapeutic Approach	Field/Nanoparticle Strategy	Proposed/Observed Stroke-Relevant Effects	Evidence Model/System	Reference
Pulsed electromagnetic fields (PEMFs)	PEMF stimulation post-ischemia	Reduces infarct size; attenuates inflammation; supports neuronal survival	Mouse cerebral ischemia model	[115]
Electromagnetic fields as stroke therapy	PEMF and other EMF paradigms	Neuroprotection via anti-inflammatory effects, angiogenesis support, and improved recovery signals	Multiple model types	[114]
MF-driven mitochondria-targeted nano-therapy	MF–driven ceria nanosystems	Targets mitochondrial dysfunction; improves tissue protection and repair endpoints in ischemia	Ischemic stroke therapeutic nanoplatform	[117]
Neuroprotection at the ischemic penumbra	Drug-loaded MNP delivery systems	Recanalization indices, thrombosis reduction, hemorrhagic risk markers, and infarct metrics	An animal model of cerebral stroke	[119]

**Table 4:** **MFs and MNPs as Neurotherapeutics in GBM.** The table below summarizes the roles of MFs and MNPs as neurotherapeutics for GBM, with a focus on overcoming resistance and improving survival outcomes associated with this aggressive brain tumor.

Neurotherapeutic Approach	Field/Nanoparticle Strategy	Proposed/Observed GBM-Relevant Effects	Evidence Model/System	Reference
Tumor Treating Fields (TTFields)	Alternating electric fields delivered via scalp arrays	Prolongs progression-free survival and overall survival in appropriately selected patients	Clinical evidence synthesized across trials	[128,129]
Intratumoral IONPs and AMF	Intratumoral SPION administration and external AMF, often combined with radiotherapy.	Demonstrates feasibility, safety, and survival signals when combined with radiotherapy	GBM patient studies	[130,140]
Static or low-frequency MFs	Static or low-frequency MF exposures	Modulates GBM proliferation, ROS, and signaling; affects EMT and metastasis-related phenotypes	Multiple model types	[134,135]
Magnetic targeting and guidance	Magnetic gradients steer SPION and drug conjugates or SPION and exosome hybrids toward GBM and peritumoral niches.	Aims to increase intratumoral payload (TMZ, siRNA, miRNA, and CRISPR cargos) while addressing BBB limitations	Multiple model types	[136,137]
Drug-loaded lipid MNPs	Lipid–magnetic constructs carrying TMZ or other agents and AMF-triggered heating and release	Enhanced tumor kill, controllable release under AMF, and combo therapy in one platform	GBM cells and in vivo model	[132,141]

## Data Availability

Data will be made available on request.
